# Maternal PCOS status and metformin in pregnancy: Steroid hormones in 5–10 years old children from the PregMet randomized controlled study

**DOI:** 10.1371/journal.pone.0257186

**Published:** 2021-09-09

**Authors:** Liv Guro Engen Hanem, Øyvind Salvesen, André Madsen, Jørn V. Sagen, Gunnar Mellgren, Petur Benedikt Juliusson, Sven Magnus Carlsen, Eszter Vanky, Rønnaug Ødegård

**Affiliations:** 1 Department of Clinical and Molecular Medicine, Faculty of Medicine and Health Sciences, Norwegian University of Science and Technology, Trondheim, Norway; 2 Children’s clinic, St. Olavs Hospital, Trondheim University Hospital, Trondheim, Norway; 3 Department of Public Health and Nursing, Faculty of Medicine and Health Sciences, Norwegian University of Science and Technology, Trondheim, Norway; 4 Hormone Laboratory, Haukeland University Hospital, Bergen, Norway; 5 Department of Clinical Science, University of Bergen, Bergen, Norway; 6 Department of Pediatrics, Haukeland University Hospital, Bergen, Norway; 7 KG Jebsen Centre for Diabetes Research, University of Bergen, Bergen, Norway; 8 Department of Health Registries, Norwegian Institute of Public Health, Bergen, Norway; 9 Department of Endocrinology, St. Olavs Hospital, Trondheim University Hospital, Trondheim, Norway; 10 Department of Obstetrics and Gynecology, St. Olavs Hospital, Trondheim University Hospital, Trondheim, Norway; 11 Centre for Obesity Research, Dept. of Surgery St. Olav University Hospital, Trondheim, Norway; Pennington Biomedical Research Center, UNITED STATES

## Abstract

**Objective:**

Polycystic ovary syndrome (PCOS) is a common endocrine disorder, with potential effects on offspring both genetically and through altered intrauterine environment. Metformin, which ameliorate hormonal disturbances in non-pregnant women with PCOS is increasingly used in pregnancy. It passes the placenta, and the evidence on potential consequences for offspring endocrine development is scarce. We explore the potential effects of maternal PCOS status and intrauterine metformin exposure on offspring steroid hormone levels.

**Design:**

This is a follow-up study of 5–10 years old children from the PregMet-study–a randomized controlled trial comparing metformin (2000 mg/day) to placebo during PCOS pregnancies. Of the 255 children invited, 117 (46%) were included.

**Methods:**

There was no intervention in this follow-up study. Outcomes were serum levels of androstenedione, testosterone, SHBG, cortisol, 17-hydroxyprogesterone, 11-deoxycortisol and calculated free testosterone converted to gender-and age adjusted z-scores from a Norwegian reference population. These were compared in i) placebo-exposed children versus children from the reference population (z-score zero) by the deviation in z-score by one-sample t-tests and ii) metformin versus placebo-exposed children by two-sample t-tests. Holm-Bonferroni adjustments were performed to account for multiple endpoints.

**Results:**

Girls of mothers with PCOS (n = 30) had higher mean z-scores of androstenedione (0.73 (95% confidence interval (CI) 0.41 to 1.06), p<0.0001), testosterone (0.76 (0.51 to 1.00), p<0.0001), and free testosterone (0.99 (0.67 to 1.32), p<0.0001) than the reference population. Metformin-exposed boys (n = 31) tended to have higher 11-deoxycortisol z-score than placebo-exposed boys (n = 24) (mean difference 0.65 (95% CI 0.14–1.17), p = 0.014).

**Conclusion:**

Maternal PCOS status was associated with elevated androgens in 5- to 10-year-old daughters, which might indicate earlier maturation and increased risk of developing PCOS. An impact of metformin in pregnancy on steroidogenesis in children born to mothers with PCOS cannot be excluded. Our findings need confirmation in studies that include participants that have entered puberty.

## Introduction

Polycystic ovary syndrome (PCOS) is a common endocrine disorder among women in fertile age [1]. Central in the etiology are insulin resistance and insulin-induced hyperandrogenism. PCOS implies increased risk of pregnancy complications, and probably an altered intrauterine environment, with potential long-term consequences for the offspring [2, 3]. There is growing evidence of increased cardiometabolic risk factors in children of women with PCOS [4–7]. Also, studies have suggested earlier pubertal maturation, higher androgen levels and risk of later PCOS among their daughters, while others found no endocrine alterations in these children [8–13]. In our previous follow-up study of the PregMet-studies, where pregnant women with PCOS were randomized to metformin or placebo, the placebo exposed offspring had higher waist circumference compared to a national reference population, whereas those exposed to metformin in addition had higher body mass index (BMI) and increased prevalence of obesity [14, 15]. More knowledge about the effects of maternal PCOS is needed to prevent the long-term health challenges in the offspring.

Metformin tends to ameliorate hormonal disturbances in non-pregnant women with PCOS [16, 17]. It has therefore been used to improve pregnancy outcomes in PCOS [18], and was recently found to decrease the prevalence of late miscarriage and pre-term births in a large individual patient data meta-analysis [19]. Two systematic reviews and meta-analyses on metformin vs placebo or insulin in pregnancy found a higher weight among metformin exposed children [20, 21]. High BMI is in general associated with earlier pubertal maturation [22–24]. There is limited knowledge about how metformin *in utero* might impact hormonal levels and pubertal maturation; except for higher free testosterone among metformin-exposed boys, metformin did not associate with any endocrine alterations in newborns from the PregMet-study [25]. In a study by Tertti et al., metformin treatment of gestational diabetes did not affect testicular size in a follow-up of 5-year-old boys [26].

In this 5–10 year follow-up of the PregMet-study, we assessed adrenal, gonadal and adipose tissue steroid hormones related to PCOS, in addition to hepatic sex-hormone-binding globulin (SHBG), which is inversely associated with obesity and insulin resistance; and calculated free testosterone [27–29]. We explore the potential effects of maternal PCOS status and intrauterine metformin exposure on offspring steroid hormone levels by comparing steroid hormones of i) children born to PCOS mothers and children from a Norwegian reference population, and ii) metformin-exposed and placebo-exposed children born to women with PCOS.

## Materials and method

The present paper represents secondary analyses of the PedMet-study: a follow-up of children born in “the Metformin in Pregnant PCOS women study” (the PregMet-study) which was a double blind, randomized, placebo-controlled study. The PedMet Clinical Trial Registration: ClinicalTrials.gov number NCT03259919. The PregMet Clinical Trial Registration: ClinicalTrials.gov number NCT00159536.

The Committee for Medical Research Ethics of Health Region IV, Norway, approved both the PregMet-study (project number 145.04) and the follow-up study (project number 2014/96). The declaration of Helsinki and the Good Clinical Practice guidelines were followed throughout the studies. The Consort 2010 statement and checklist has been followed when reporting, as appropriate. Written, informed consent was obtained from each child’s parent or guardian before inclusion.

The PregMet-study: Between 2005 and 2009, 257 pregnant women aged 18–45 years, with PCOS according to the Rotterdam criteria were included with 274 singleton pregnancies at 5–12 weeks of gestation, at 11 study centers in Norway. Seventeen women participated twice[30]. Participants were randomized to metformin (2000 mg daily) or placebo throughout pregnancy. An intake of >85% of the tablets was self-reported in 80% of the participants. Details of design, objectives, and results are described elsewhere [18].

The follow-up study–The PedMet-study: Of the 274 pregnancies in the PregMet-study, 255 children were eligible, and their parent or guardian were asked to their permission to participate.

Participating children were included at St. Olavs University Hospital in Trondheim and nine additional study centers in Norway by trained medical staff employed at the Norwegian University of Science and Technology (NTNU) in Trondheim. Mothers were informed of their allocation after the last delivery in the PregMet-study, but were requested not to inform study staff, who were blinded for group allocation during data collection.

Information on sex, age, ethnicity, presence or history of body odor or acne, and a general medical history was obtained by standardized interviewer-administered questionnaires. Tanner stage was determined either by direct evaluation, or by the parent/guardian in accordance with the illustrations of the Tanner scale [31].

Height was measured with a Seca stadiometer. Head circumference was measured over the most prominent part of the occiput, and just above the supraorbital ridge with a measuring tape. Waist circumference was measured at the minimal waist with a measuring tape. Total body weight was measured on an InBody 720 (BIOSPACE, Korea) in children included at St. Olavs University Hospital. The 43 children included at other study sites were weighed on a digital weighing scale. Body Mass Index (BMI) was calculated from the formula: weight (kg) / height (m)^2^. As the age at inclusion of the children varied, all anthropometric measurements were converted to z-scores according to gender and age from a Norwegian reference population, the Bergen growth Study 1 [32, 33]. Blood pressure and heart rate were measured 3 times after >10 minutes seated rest, >2 minutes apart. The mean of the two last measurements was included in the statistical analysis.

### Biochemical analyses

Blood samples were drawn from an antecubital vein between 0800 and 1100 h after an overnight fast. Serum was stored at -80°C and thawed once before analysis. Androstenedione, testosterone, cortisol, 17-hydroxyprogesterone and 11-deoxycortisol were analyzed by liquid chromatography-tandem mass spectrometry (LC-MS/MS) at the Hormone Laboratory, Haukeland University Hospital, Bergen, Norway. The LC-MS/MS analyses were accredited by Norsk Akkreditering 14.12.2018 (NS-EN ISO 15189 (2012)). The inter-assay coefficients of variation were < 20% for all steroid hormones analyzed. The lower limits of quantification were 0.02 nmol/l for testosterone, 0.12 nmol/l for androstenedione, 0.24 nmol/l for 17-hydroxyprogesterone, 0.1 nmol/l for 11-deoxycortiol and 1.95 nmol/l for cortisol [34].

In the PedMet-study, sex hormone binding globulin (SHBG) was analyzed at the Hormone Laboratory, Oslo University Hospital, Oslo, Norway by non-competitive immunoluminometric assay (ILMA). Free testosterone index was calculated as the percentage of total testosterone divided by SHBG.

Hormone z-scores were interpolated from the “Bergen Growth Study 2” references for male [35] and female [36] hormones rendered by the generalized additive model for location, scale and shape (GAMLSS) package in R (R Core Team, Vienna, Austria). Briefly, the LMS method for normalized growth standards [37] enables the calculation of z-scores for new observations in relation to the smooth (L), mean (M) and coefficient of variation (S) curves. For the current study, all z-scores therefore represent the sex- and age-adjusted equivalents of the indicated measurement, in relation to the previously described population samples of healthy 6- to 16 year-old Norwegian children [24, 38, 39].

Blood samples of control subjects from the Bergen Growth Study 2 were analyzed at the Hormone Laboratory, Haukeland University Hospital, Bergen. Steroid hormones were analyzed by LC-MS/MS, as described above, and SHBG by chemiluminescent immunoassay (CLIA) on Immulite 2000xpi kit (Siemens Healthineers, Germany) [34].

### Statistical analyses

Data entry, management and analyses were performed at the Department of Clinical and Molecular Medicine at the Norwegian University of Science and Technology. The impact of maternal PCOS on steroid hormones and SHBG was examined by assessing the deviation in z-scores between the placebo group and the reference population (z-score zero), by one-sample t-tests. To compare the BMI and the waist circumference z-scores in the placebo group and controls from the Bergen Growth Study 2, independent samples t-tests were performed. Differences between the placebo and metformin groups of steroid hormones and SHBG z-scores, maternal baseline characteristics, pregnancy outcomes and anthropometric measurements were analyzed on IBM SPSS Statistics version 22.0 (IBM, SPSS inc USA, Chicago IL) by a two-sample t-test for continuous variables and a chi-square or Fishers exact test for categorical variables. To estimate the potential effects of maternal PCOS and metformin on offspring hormones *not mediated through changes in BMI*, maternal PCOS and metformin effects on hormone z-scores were adjusted for offspring BMI z-scores, by linear regression analyses. Subgroup analyses according to gender were performed, as the levels of steroid hormones vary between sexes also before puberty, and as both maternal PCOS and metformin in pregnancy might affect steroid hormones differently according to sex [40]. Multiple primary endpoints—hormone z-scores, Tanner stage and signs of puberty—were adjusted for by the Holm-Bonferroni procedure, with a family-wise error rate at 5% [41, 42]. Also Insulin like Growth Factor-1, DHEAS, Anti Mullerian Hormone, fT4 and TSH were measured in children from the PregMet study, but as they have not been analyzed in the Bergen Growth Study 2, they could neither be converted to z-scores, nor age-adjusted and are therefore less accurate, and were not included in the final analyses.

## Results

**Fig 1:**
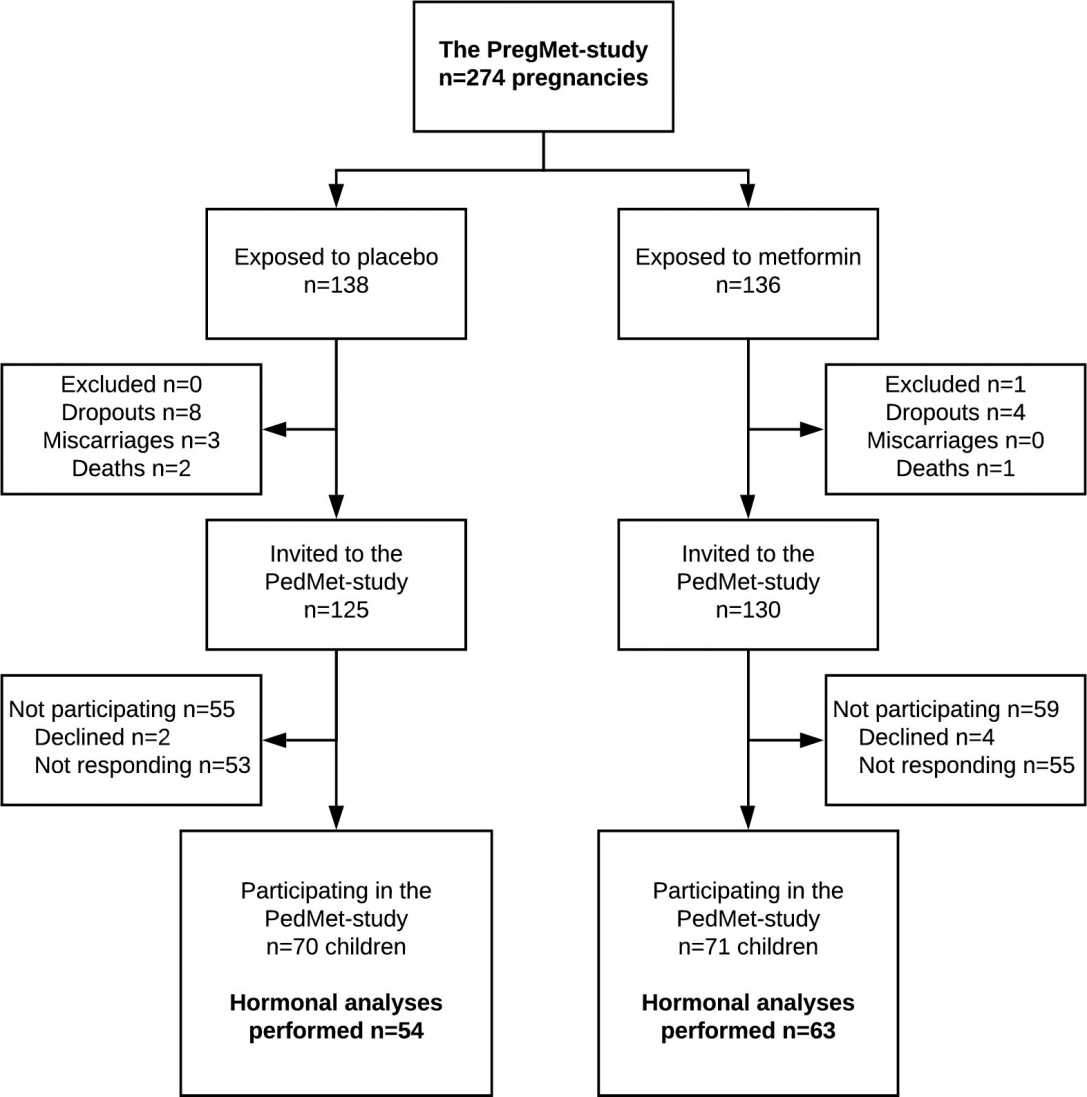
Flow chart of the PregMet and PedMet studies.

From April 2014 to July 2016, we included 141 children in the PedMet-study. Blood samples were drawn, and steroid hormone levels determined in 117 (46%) of the invited children (Fig 1). The participation rate was 48% in the metformin group, and 43% in the placebo group (p = 0.29).

Maternal baseline characteristics, mode of conception, metformin use at conception, pregnancy outcomes, birth anthropometrics and breastfeeding in participants vs non-participants and in the treatment groups are presented in S1–S3 Tables, and were comparable between groups.

The mean age of the children at follow-up was 7.4 years ± 1.2 SD (Table 1).

**Table 1:** Age, Tanner stage and signs of puberty in the children (n = 117) at inclusion at 5–10 years of age.

	Children of both sexes		Boys		Girls	
	Placebo,	Metformin, n = 63	Placebo,	Metformin, n = 31	Placebo,	Metformin, n = 32
	n = 54		n = 24		n = 30	
Age, years	7.46 ± 1.18	7.40 ± 1.21	7.54 ± 1.22	7.56 ± 1.23	7.40 ± 1.16	7.23 ± 1.219
Tanner stage, n						
I	50 (93)	61 (96)	22 (92)	31 (100)	28 (93)	30 (94)
II	4 (7)	1 (2)	2 (8)	0 (0)	2 (7)	1 (3)
III	0 (0)	1 (2)	0 (0)	0 (0)	0 (0)	1 (3)
Body odor, n	6 (11)	10 (16)	2 (8)	5 (16)	4 (13)	5 (16)
Acne/oily skin, n	2 (4)	3 (5)	0 (0)	1 (3)	2 (7)	2 (6)

Data presented as mean ± Standard Deviation or numbers (%) as appropriate.

None of the comparisons had a p-value under 0.05.

### The maternal PCOS effect

The impact of maternal PCOS on steroid hormones was examined by assessing the deviation in z-scores between the placebo-exposed children and children from the Bergen Growth Study 2 (z-score zero).

Children of mothers with PCOS (boys and girls) had significantly higher mean z-score of androstenedione (0.53 (95% confidence interval (CI) 0.27 to 0.79) p = 0.0001), testosterone (0.53 (95% CI 0.29 to 0.77), p<0.0001), and free testosterone (0.67 (95% CI 0.38 to 0.96), p<0.0001) than children from the Bergen Growth Study 2 (Table 2A). There was a tendency towards higher cortisol (0.34 (95% CI 0.08 to 0.61), p = 0.012) and 17-hydroxyprogesterone (0.42 (95% CI 0.15 to 0.69), p = 0.003), but the difference was not significant after Holm-Bonferroni adjustment.

**Table 2:** Steroid hormone z-scores in children from the placebo and metformin groups.

**a) Children of both sexes**					
	The maternal PCOS effect^a^			The metformin effect^b^	
	Placebo, n = 54	p	Metformin, n = 63	Δ M-P (95% CI)	p
	Mean (95% CI)		Mean (95% CI)		
Androstenedione z-score	0.53 (0.27 to 0.79)	.0001*	0.66 (0.41 to 0.91)	0.13 (-0.23 to 0.49)	.470
Testosterone z-score	0.53 (0.29 to 0.77)	< .0001*	0.53 (0.30 to 0.76)	0.00 (-0.33 to 0.33)	.995
SHBG z-score	-0.28 (-0.55 to 0.00)	.051	-0.28 (-0.51 to -0.05)	0.01 (-0.36 to 0.34)	.973
Cortisol z-score	0.34 (0.08 to 0.61)	.012	0.69 (0.42 to 0.96)	0.35 (-0.03 to 0.72)	.070
17-OH-progesterone z-score	0.42 (0.15 to 0.69)	.003	0.83 (0.62 to 1.04)	0.41 (0.08 to 0.74)	.015
11-deoxycortisol z-score	0.13 (-0.11 to 0.37)	.269	0.46 (0.22 to 0.69)	0.32 (-0.01 to 0.66)	.056
Free testosterone z-score	0.67 (0.38 to 0.96)	< .0001*	0.63 (0.35 to 0.91)	-0.04(-0.44 to 0.36)	.841
**b) Boys**					
	The maternal PCOS effect^a^			The metformin effect^b^	
	Placebo, n = 24	p	Metformin, n = 31	Δ M-P (95% CI)	p
	Mean (95% CI)		Mean (95% CI)		
Androstenedione z-score	0.27 (-0.15 to 0.69)	.195	0.74 (0.45 to 1.02)	0.46 (-0.02 to 0.95)	.058
Testosterone z-score	0.25 (-0.20 to 0.70)	.264	0.54 (0.22 to 0.85)	0.29 (-0.23 to 0.81)	.268
SHBG z-score	0.03 (-0.45 to 0.51)	.905	-0.43 (-0.75 to -0.11)	-0.46 (-0.99 to 0.08)	.096
Cortisol z-score	0.51 (0.17 to 0.84)	.005	0.82 (0.37 to 1.27)	0.31 (-0.27 to 0.89)	.284
17-OH-progesterone z-score	0.40 (-0.07 to 0.87)	.089	0.93 (0.61 to 1.24)	0.53 (-0.00 to 1.06)	.052
11-deoxycortisol z-score	0.08 (-0.31 to 0.47)	.670	0.74 (0.38 to 1.09)	0.65 (0.14 to 1.17)	.014
Free testosterone z-score	0.25 (-0.23 to 0.73)	.292	0.67 (0.34 to 1.00)	0.42 (-0.13 to 0.97)	.133
**c) Girls**					
	The maternal PCOS effect^a^			The metformin effect^b^	
	Placebo, n = 30	p	Metformin, n = 32 Mean (95% CI)	Δ M-P (95% CI)	p
	Mean (95% CI)				
Androstenedione z-score	0.73 (0.41 to 1.06)	< .0001*	0.59 (0.16 to 1.01)	-0.15 (-0.68 to 0.38)	.580
Testosterone z-score	0.76 (0.51 to 1.00)	< .0001*	0.53 (0.18 to 0.88)	-0.23 (-0.66 to 0.19)	.280
SHBG z-score	-0.51(-0.83 to-0.19)	.003	-0.13 (-0.47 to 0.20)	0.37 (-0.08 to 0.83)	.104
Cortisol z-score	0.21 (-0.19 to 0.61)	.291	0.56 (0.24 to 0.89)	0.35 (-0.15 to 0.86)	.165
17-OH-progesterone z-score	0.44 (0.11 to 0.76)	.010	0.74 (0.44 to 1.03)	0.30 (-0.13 to 0.73)	.162
11-deoxycortisol z-score	0.18 (-0.14 to 0.49)	.291	0.19 (-0.11 to 0.48)	0.01 (-0.41 to 0.44)	.952
Free testosterone z-score	0.99 (0.67 to 1.32)	< .0001*	0.59 (0.12 to 1.06)	-0.40 (-0.96 to 0.16)	.157

*p-values statistically significant after Holm-Bonferroni adjustment.

^a^The impact of maternal PCOS on steroid hormones was assessed by the deviation in z-scores between the placebo group and the reference population (z-score zero) by one sample t-tests.

^b^Δ M-P expresses the metformin effect on hormone mean z-scores, assessed by two sample t-tests.

All z-scores were calculated from the Bergen Growth Study 2 references, according to gender and age.

CI: confidence interval; SHBG: sex hormone binding globulin.

Hormone levels not converted to z-scores are presented in the S4 Table.

In boys born to women with PCOS, there was a non-significant higher mean cortisol z-score (0.51 (95% CI 0.17 to 0.84), p = 0.005) after Holm-Bonferroni adjustment, and otherwise no difference in hormone levels compared to boys from the Bergen Growth Study 2 (Table 2B).

Girls of mothers with PCOS had higher mean z-score of androstenedione (0.73 (95% CI 0.41 to 1.06), p<0.0001) testosterone (0.76 (95% CI 0.51 to 1.00), p<0.0001) and free testosterone (0.99 (95% CI 0.67 to 1.32), p<0.0001), than girls from the Bergen Growth Study 2 (Table 2C). There was a non-significant higher 17-hydroxyprogesterone z-score (0.44 (95% CI 0.11 to 0.76), p = 0.01), and lower of SHBG z-score (-0.51 (95% CI -0.83 to-0.19), p = 0.003), after Holm-Bonferroni adjustments. Adjustment for the BMI z-scores of the children had little impact on the estimated effect of maternal PCOS on the hormone z-scores in children (S5 Table). Additional adjustment for offspring waist circumference z-scores did not change the effect estimates (data not shown).

Children born to women with PCOS tended to have higher mean BMI z-scores (mean difference 0.40 (95% CI 0.13 to 0.68), p = 0.004, and waist circumference z-scores (mean difference: 0.74, 95% CI 0.56 to 0.92, p = 0.0001) than children in the Bergen Growth Study 2. In boys, the mean difference in BMI z-score was 0.27, (95% CI -0.11 to 0.65), p = 0.168, while the mean difference in waist circumference z-score was 0.72 (95% CI 0.47 to 0.98), p = 0.0001. In girls, the mean difference in BMI z-score was 0.51 (95% CI 0.13 to 0.90), p = 0.009, and the mean difference in waist circumference z-score was 0.75 (95% CI 0.49 to 1.02), p = 0.0001.

### The metformin effect

There was a non-significant higher mean 17-hydroxyprogesterone z-score in the metformin group than in the placebo group (mean difference 0.41 (95% CI 0.08 to 0.74), p = 0.015), and otherwise no difference when assessing both sexes, after Holm-Bonferroni adjustment (Table 2A).

Metformin-exposed boys had non-significant higher 11-deoxycortisol z-score than placebo exposed boys (mean difference 0.65 (95% CI 0.14 to 1.17), p = 0.014, after Holm-Bonferroni adjustment. Other hormones did not differ between metformin and placebo-exposed boys (Table 2B).

There were no difference in hormone levels between metformin- and placebo-exposed girls (Table 2C). Adjusting for BMI z-scores in the children had little impact on the estimated effect of metformin on hormone z-scores in the children (S6 Table). Additional adjustment for offspring waist circumference z-scores also had little effect on the estimated effect (data not shown).

Most children had Tanner stage 1, and no signs of puberty. There was no difference in distribution of Tanner stages and signs of puberty between the groups (Table 1). As previously published, there was a tendency towards higher waist circumference z-score and waist-to-height ratio z-score in metformin vs placebo exposed boys (Table 3) [14].

**Table 3:** Anthropometrics and measures of glucose homeostasis, all children (n = 122) at inclusion, and according to gender.

	Children of both sexes			Boys			Girls		
	Placebo	Metformin	p	Placebo	Metformin	p	Placebo	Metformin	p
	N = 54	N = 63		N = 24	N = 31		N = 30	N = 32	
Anthropometrics									
BMI z-score	0.26 (-0.03 to 0.54)	0.53 (0.22 to 0.84)	.20	0.10 (-0.31 to 0.45)	0.60 (0.15 to 1.04)	.10	0.38 (-0.05 to 0.81)	0.46 (0.01 to 0.93)	.78
Weight z-score	0.23 (-0.10 to 0.55)	0.51 (0.22 to 0.81)	.19	0.11 (-0.34 to 0.57)	0.63 (0.20 to 1.06)	.10	0.32 (-0.16 to 0.79)	0.39 (-0.04 to 0.83)	.81
Height z-score	0.09 (-0.21 to 0.40)	0.18 (-0.07 to 0.43)	.68	0.13 (-0.36 to 0.62)	0.35 (-0.05 to 0.74)	.47	0.07 (-0.34 to 0.48)	-0.00 (-0.31 to 0.31)	.79
WC z-score	0.51 (0.27 to 0.74)	0.81 (0.56 to 1.07)	.09	0.34 (0.08 to 0.60)	0.91 (0.55 to 1.26)	.02	0.64 (0.26 to 1.02)	0.72 (0.33 to 1.11)	.76
WHtR z-score	0.47 (0.26 to 0.69)	0.82 (0.56 to 1.07)	.06	0.28 (0.01 to 0.55)	0.84 (0.48 to 1.20)	.02	0.67 (0.33 to 1.00)	0.79 (0.39 to 1.19)	.63
Glucose homeostasis measures									
Insulin C-peptide	0.30 ± 0.17	0.32 ± 0.22	.61	0.29 ± 0.18	0.36 ± 0.29	.28	0.31 ± 0.16	0.28 ± 0.13	.42
HbA1c	5.05 ± 0.22	5.05 ± 0.18	.67	5.08 ± 0.23	5.12 ± 0.17	.46	5.03 ± 0.22	5.01 ± 0.18	.81
Fasting glucose	4.75 ± 0.45	4.71 ± 0.49	.65	4.77 ± 0.44	4.80 ± 0.54	.87	4.74 ± 0.49	4.63 ± 0.44	.41

Data presented as mean (95% Confidence Interval) or mean ± Standard Deviation as appropriate.

BMI: body mass index, calculated as height/meter^2^, WC = waist circumference; WHtR: waist-to-height ratio.

These data represent a subgroup of previously published data [15].

## Discussion

In this study, daughters of women with PCOS had increased levels of androgens compared to the reference population. Boys exposed to metformin *in utero* tended to have higher levels of 11-deoxycortisol.

### The maternal PCOS effect

The possible altered steroidogenesis in daughters of women with PCOS, as suggested by our findings of higher androgens, might be due to effects on adrenal glands, ovaries or fat tissue. Tendencies towards a lower SHBG z-score and a significantly increased free testosterone z-score was observed among girls [28], and androstenedione z-score, which normally increases in early puberty, was higher in PCOS children than controls [43]. In puberty, SHBG levels tend to decrease, while androgens tend to increase, both indicating an increased risk of earlier puberty among PCOS offspring [27, 28, 44]. An earlier adrenal or gonadal maturation in daughters of women with PCOS can therefore not be excluded. The BMI and waist circumference of the children born to women with PCOS was higher than in the Bergen Growth Study 2. In general, an increased BMI in children of this age is related to earlier adrenal and gonadal maturation, while adiposity in girls is related to higher levels of testosterone and lower levels of SHBG, in part due to hyperinsulinemia. Some of the differences in hormones between the groups could therefore relate to the increased central adiposity in this population [23, 28], but adjusting for BMI and waist circumference z-scores had limited impact on the effect estimates. Measures of glucose homeostasis were not available in the Bergen Growth Study 2 for comparison.

Stimulation tests with GnRH agonists have shown that ovaries are the source of hyperandrogenism in most adult women with PCOS [45]. About 50% of women with PCOS have, however, increased levels of adrenal androgens, and Maliqueo et al. found that daughters of women with PCOS more often had exaggerated adrenarche and earlier bone maturation than controls [9]. Signs of early pubertal maturation, higher prevalence of PCOM, higher levels of AMH, testosterone, 17-hydroxyprogesterone, DHEAS and lower SHBG have also been described among daughters of women with PCOS [8–11, 46–48]. Also in sons of women with PCOS, hormonal characteristics of early pubertal maturation have been described including higher FSH, androstenedione levels, AMH and bioavailable testosterone, lower SHBG and testicular volume, but with sperm count comparable to controls [49, 50]. Other studies have reported no alterations in androgens between PCOS offspring and controls, and the diverging results are probably due to small study samples and heterogeneity in terms of study design and population selection [10, 12, 13]. The strongest evidence is, however, in accordance with the present results of higher androgens and earlier pubertal maturation among daughters of women with PCOS.

### The metformin effect

There were tendencies towards higher levels of 11-deoxycortisol (boys only) and 17-hydroxyprogesterone (total sample) in the metformin group. 11-deoxycortisol is a glucocorticoid and mainly a precursor of the more potent cortisol, but might also be converted to androstenedione [51]. 17-hydroxyprogesterone is a progestogen, and a precursor of 11-deoxycortisol and androstenedione [52]. We have previously published, and observed also in the present sub-population, tendencies of increased BMI and waist circumference in the metformin group [14]. Increased levels of cortisol, of which 11-deoxycortisol is a precursor, have repeatedly been associated to overweight, irrespective of age, but the mechanism behind remains unexplained [53]. Some impact on steroid hormones and SHBG could be expected from the trend towards higher measures of adiposity in the metformin group. Adjusting for BMI and waist circumference z-scores had, however, little impact on the effect estimates of metformin. How metformin in utero potentially might impact these steroid precursors independently of BMI is not known, and the clinical significance of this finding is uncertain.

Two systematic reviews and meta-analyses of RCTs on metformin vs placebo/insulin in PCOS pregnancies or in gestational diabetes mellitus supports a higher weight among metformin exposed children, which in turn might impact the timing of puberty [20, 21, 23]. The evidence on how metformin *in utero* might influence hormonal levels and maturation in childhood is essentially lacking. While mice exposed to metformin *in utero* had reduced testicular size, and human fetal gonads exposed to metformin *in vitro* had reduced testosterone secretion [54], no association was found between metformin and testicular size of 52 children at ~5 years of age, after their mothers were randomized to metformin or insulin for the treatment of gestational diabetes mellitus [26]. In the present study, the metformin and placebo groups did not differ in DHEAS, androstenedione levels or in signs of clinical androgen action. However, whether metformin, or the increased central adiposity related to it, impact pubertal development is uncertain based on our findings, as the mean age was only 7.5 years, and as few of the children had reached puberty.

Based on the present results, an effect of metformin on offspring steroid hormones cannot be excluded. The results from studies where metformin was used in PCOS pregnancies imply a particularly cautious follow up of these children in order to prevent overweight and the associated risk of early puberty [14, 15, 23, 24].

An obvious strength of this study is the study design—a follow-up of a randomized placebo-controlled, double blind study: the PregMet-study. The metformin and placebo groups were comparable at baseline. The PregMet-study population represented a broad spectrum of women with *known* PCOS diagnosis and good compliance to study medication. It is uncertain whether the results of this study are applicable to women without PCOS, without *known* PCOS, or to women who differ from the PregMet-population in ethnicity, BMI or other characteristics. Both the PregMet and the PedMet-studies adhered to strict methodology, and examinations were performed by trained study personnel. Steroid hormone analyses were performed by LC-MS/MS, which, compared to immunoassays, offers superior analytical specificity, sensitivity and accuracy, even at low concentrations. The reference population consists of a large random sample of Norwegian children.

This study has some limitations. The follow-up rate is low: 46%. However, maternal baseline characteristics were comparable between participants and non-participants. The low follow-up rate, combined with small numbers, multiple tests and subgroup analyses increases the risk of random errors and false positive findings. Holm-Bonferroni adjustments were performed to account for this.

## Conclusion

Elevated androgen levels were present in preadolescent daughters of women with PCOS, indicating higher risk of early pubertal maturation. Metformin exposed boys tended to have higher levels of 11-deoxycortisol compared to placebo exposed boys, and a modifying effect on steroidogenesis of metformin exposure in utero cannot be excluded. Our findings need to be confirmed by studies on offspring who have entered puberty.

## Supporting information

S1 Table Maternal characteristics early in pregnancy at inclusion, in all participants and according to gender. (DOCX)

S2 Table Pregnancy outcomes, birth anthropometrics and breastfeeding in all pregnancies and according to gender. (DOCX)

S3 Table Maternal characteristics early in pregnancy at inclusion in the PregMet-study, pregnancy outcomes, birth anthropometrics and breastfeeding in participants and non-participants. (DOCX)

S4 Table Hormonal levels in placebo and metformin exposed children including children of both sexes, boys and girls. (DOCX)

S5 Table Effect of maternal PCOS on steroid hormones in children, unadjusted, and adjusted for children BMI z-score. (DOCX)

S6 Table Metformin effect on steroid hormones in children from the PregMet study, unadjusted, and adjusted for BMI z-score. (DOCX)
