# Supplementary material for: Maternal PCOS status and metformin in pregnancy: Steroid hormones in 5–10 years old children from the PregMet randomized controlled study
Source: PLoS One. 2021 Sep 9;16(9):e0257186. doi: 10.1371/journal.pone.0257186 (PMC8428669; doi:10.1371/journal.pone.0257186)
Supplement: S1 Table — (DOCX) [file pone.0257186.s001.docx]

|  |  |  |  |  |  |  |  |  |  |  |
| --- | --- | --- | --- | --- | --- | --- | --- | --- | --- | --- |
|  |  | **All** | | | **Boys** | | | **Girls** | | |
|  |  | Placebo, n=54 | Metformin, n=63 | p | Placebo, n=25 | Metformin, n=32 | p | Placebo, n=30 | Metformin, n=35 | p |
| Age (years) |  | 30.5 ± 4.1 | 29.3 ± 3.5 | .267 | 29.4 ± 4.1 | 29.5 ± 3.2 | .925 | 31.4 ± 4.0 | 29.7 ± 4.4 | .114 |
| Height (cm) |  | 168.4 ± 5.3 | 166.9 ± 5.6 | .536 | 168.4 ± 5.2 | 167.3 ± 6.6 | .517 | 168.3 ± 5.5 | 167.2 ± 4.9 | .397 |
| Weight (kg) |  | 81.7± 18.9 | 81.4 ± 19.5 | .591 | 78.2 ± 17.0 | 81.0 ± 20.9 | .582 | 83.9 ± 20.4 | 81.2 ± 18.9 | .575 |
| BMI (kg/m²) |  | 28.8 ± 6.6 | 29.2 ± 7.0 | .555 | 27.7 ± 6.4 | 28.9 ±6.9 | .500 | 29.6 ± 6.7 | 29.11 ± 7.2 | .767 |
| Systolic blood pressure (mmHg) |  | 119 ± 12 | 119 ± 12 | .723 | 113 ± 10 | 118 ± 11 | .147 | 123 ± 12 | 119 ± 12 | .216 |
| Diastolic blood pressure (mmHg) |  | 74 ± 10 | 74 ± 9 | .720 | 70 ± 7 | 73 ± 11 | .202 | 76 ± 11 | 73 ± 8 | .120 |
| Heart rate (bpm)^b^ |  | 73 ± 9 | 74 ± 11 | .451 | 72 ± 10 | 74 ± 11 | .499 | 73 ± 9 | 75 ± 11 | .565 |
| OGTT (75g) |  |  |  |  |  |  |  |  |  |  |
| Fasting plasma glucose (mmol/L) |  | 4.6 ± 0.6 | 4.6 ± 0.5 | .575 | 4.5 ±0.5 | 4.6 ± 0.5 | .769 | 4.8 ± 0.6 | 4.7 ± 0.5 | .601 |
| 2 h plasma glucose (mmol/L)^c^ |  | 5.3 ± 1.6 | 5.3 ± 1.6 | .851 | 4.7 ± 1.0 | 5.2 ± 1.3 | .107 | 5.7 ± 1.9 | 5.3 ± 1.7 | .367 |
| Total cholesterol (mmol/L)^d^ |  | 4.4 ± 0.7 | 4.8 ± 1.1 | .083 | 4.4 ± 0.7 | 4.9 ± 1.3 | .053 | 4.4 ± 0.8 | 4.8 ± 0.9 | .136 |
| HDL cholesterol (mmol/L)^e^ |  | 1.5 ± 0.3 | 1.6 ± 0.3 | .514 | 1.6 ± 0.3 | 1.6 ± 0.4 | .876 | 1.5 ± 0.4 | 1.7 ± 0.3 | .054 |
| Triglycerides (mmol/L)^e^ |  | 1.1 ± 0.6 | 1.1 ± 0.4 | .240 | 0.9 ± 0.4 | 1.2 ± 0.5 | .024 | 1.3 ± 0.6 | 1.1 ± 0.4 | .071 |
| Smoking |  | 4 (7.3) | 4 (6.0) | .772 | 1 (4.0) | 0 (0.0) | .254 | 3 (10.0) | 4 (11.4) | .853 |
| Parity |  |  |  | .347 |  |  | .578 |  |  | .437 |
| Nullipara |  | 29 (52.7) | 41 (61.2) |  | 13 (52.0) | 19 (59.4) |  | 16 (53.3) | 22 (62.9) |  |
| Multipara |  | 26 (47.3) | 26 (38.8) |  | 12 (48.0) | 13 (40.6) |  | 14 (46.7) | 13 (37.1) |  |
| PCOS phenotype^a^ |  |  |  | .451 |  |  | .381 |  |  | .313 |
| Type I |  | 34 (61.8) | 39 (58.2) |  | 17 (68.0) | 15 (46.9) |  | 17 (56.7) | 24 (68.6) |  |
| Type II |  | 5 (9.1) | 7 (10.4) |  | 2 (8.0) | 5 (15.6) |  | 3 (10.0) | 2 (5.7) |  |
| Type III |  | 0 (0.0) | 3 (4.5) |  | 0 (0.0) | 1 (3.1) |  | 0 (0.0) | 2 (5.7) |  |
| Type IV |  | 16 (29.1) | 18 (26.9) |  | 6 (24.0) | 11 (34.4) |  | 10 (33.3) | 7 (20.0) |  |
| Metformin use at conception |  | 16 (29.1) | 25 (37.3) | .339 | 6 (24.0) | 13 (40.6) | .186 | 10 (33.3) | 12 (34.3) | .936 |
| Mode of conception |  |  |  | .912 |  |  | .274 |  |  | .208 |
| Spontaneously (%) |  | 29 (52.7) | 36 (53.7) |  | 13 (52.0) | 12 (37.5) |  | 16 (53.3) | 24 (68.6) |  |
| ART (%) |  | 26 (47.3) | 31 (46.3) |  | 12 (48.0) | 20 (62.5) |  | 14 (46.7) | 11 (31.4) |  |

S1 Table. Maternal characteristics early in pregnancy at inclusion, in all participants and according to gender

None of the comparisons between the groups showed statistical significant difference (p-value < 0.01)

Data presented as mean ± standard deviation or numbers (%) as appropriate

BMI: body mass index calculated from the formula weight (kg)/ height (m)^2^; bpm: beats per minute; OGTT: oral glucose tolerance test; HDL: High density lipoprotein; ART: assisted reproductive therapy comprises ovulation induction, In Vitro Fertilization and Intracytoplasmic Sperm Injection

^a^ PCOS phenotypes: Type 1: PCO + hyperandrogenism + oligoamenorrhea; Type II: PCO + hyperandrogenism; Type III: hyperandrogenism + oligoamenorrhea; Type IV: PCO + oligoamenorrhea

^b^Numbers in total sample: 65 meformin exposed, 53 placebo exposed, boys: 32 metformin exposed, 24 placebo exposed, girls: 33 metformin exposed, 29 placebo exposed

^c^Numbers in total sample: 67 meformin exposed, 54 placebo exposed, boys: 32 metformin exposed, 25 placebo exposed, girls: 35 metformin exposed, 29 placebo exposed

^d^Numbers in total sample: 65 meformin exposed, 52 placebo exposed, boys: 31 metformin exposed, 22 placebo exposed, girls: 34 metformin exposed, 30 placebo exposed

^e^Numbers in total sample: 65 meformin exposed, 53 placebo exposed, boys: 31 metformin exposed, 23 placebo exposed, girls: 34 metformin exposed, 30 placebo exposed
