# Supplementary material for: Maternal PCOS status and metformin in pregnancy: Steroid hormones in 5–10 years old children from the PregMet randomized controlled study
Source: PLoS One. 2021 Sep 9;16(9):e0257186. doi: 10.1371/journal.pone.0257186 (PMC8428669; doi:10.1371/journal.pone.0257186)
Supplement: S2 Table — (DOCX) [file pone.0257186.s002.docx]

|  |  |  |  |  |  |  |  |  |  |
| --- | --- | --- | --- | --- | --- | --- | --- | --- | --- |
|  | **All** | | | **Boys** | | | **Girls** | | |
|  | Placebo, n=54 | Metformin n=63 | p | Placebo, n=24 | Metformin, n=31 | p | Placebo, n=30 | Metformin, n=32 | p |
| Weight gain in pregnancy (kg) | 10.3 ± 5.2 | 9.9 ± 4.9 | .674 | 10.5 ± 5.4 | 10.9 ± 5.4 | .772 | 10.2 ± 5.0 | 8.9 ± 4.2 | .293 |
| Pregnancy complications |  |  |  |  |  |  |  |  |  |
| Preterm birth | 7 (12.9) | 3 (4.8) | .120 | 3 (12.5) | 0 (0.0) | .044 | 4 (13.3) | 3 (9.3) | .654 |
| GDM | 14 (25.9) | 14 (22.2) | .674 | 5 (20.8) | 4 (12.9) | .430 | 9 (30.0) | 10 (31.2) | .849 |
| Preeclampsia | 5 (9.3) | 5 (7.9) | .819 | 1 (4.2) | 1 (3.2) | .853 | 4 (13.3) | 4 (12.5) | .960 |
| Placenta weight (g) | 669 ± 177 | 652 ± 161 | .599 | 689 ± 167 | 678 ± 159 | .817 | 654 ± 186 | 626 ± 162 | .558 |
| Birth anthropometrics |  |  |  |  |  |  |  |  |  |
| Length z-score^a^ | -0.41 (-0.78 to -0.03) | -0.34 (-0.62 to -0.05) | .754 | -0.32 (-0.89 to 0.25) | 0.14 (-0.21 to 0.50) | .149 | -0.48 (-1.02 to -0.06) | -0.81 (-1.19 to -0.43) | .305 |
| Weight z-score^a^ | -0.50 (-0.92 to -0.08) | -0.37 (-0.67 to -0.07) | .621 | -0.45 (-1.16 to 0.27) | 0.00 (-0.39 to 0.39) | .240 | -0.54 (-1.08 to 0.00) | -0.75 (-1.18 to -0.32) | .522 |
| BMI z-score^a^ | -0.09 (-0.36 to 0.19) | -0.14 (-0.39 to 0.10) | .748 | -0.08 (-0.51 to 0.36) | -0.05 (-0.35 to 0.26) | .903 | -0.09 (-0.47 to 0.29) | -0.24 (-0.64 to 0.16) | .579 |
| Head circumference z-score^a^ | -0.16 (-0.52 to 0.19) | 0.11 (-0.17 to 0.39) | .232 | 0.22 (-0.27 to 0.70) | 0.50 (0.18 to 0.81) | .300 | -0.45 (-0.96 to 0.06) | -0.24 (-0.67 to 0.19) | .529 |
| Breastfeeding |  |  |  |  |  |  |  |  |  |
| Exclusive (months) | 4.2 ± 2.8 | 4.4 ± 2.8 | .777 | 3.6 ± 2.2 | 3.9 ± 2.8 | .706 | 4.7 ± 3.2 | 4.8 ± 2.9 | .892 |
| Total (months) | 9.1 ± 3.8 | 9.1 ± 4.3 | .996 | 8.1 ± 4.4 | 8.9 ± 4.5 | .587 | 9.9 ± 3.1 | 9.3 ± 4.2 | .591 |

S2 Table. Pregnancy outcomes, birth anthropometrics and breastfeeding in all pregnancies and according to gender

Data presented as mean ± standard deviation, numbers (%) or mean (95% Confidence Interval) as appropriate

GDM: gestational diabetes mellitus defined as fasting plasma glucose higher than 7.0 mmol/L and/or 2-h serum glucose higher than 7.8 mmol/L after an 75 g oral glucose tolerance test, BMI: body mass index calculated from the formula weight (kg)/ height (m)^2^

^a^z-scores were calculated according to gender and age from a Norwegian reference population [29,30]
