# Supplementary material for: Maternal PCOS status and metformin in pregnancy: Steroid hormones in 5–10 years old children from the PregMet randomized controlled study
Source: PLoS One. 2021 Sep 9;16(9):e0257186. doi: 10.1371/journal.pone.0257186 (PMC8428669; doi:10.1371/journal.pone.0257186)
Supplement: S3 Table — (DOCX) [file pone.0257186.s003.docx]

|  | **Participants**  **n=117** | **Non-participants**  **n=138** | **p** |
| --- | --- | --- | --- |
| Age (years) | 29.9 ± 3.9 | 29.3 ± 4.7 | .275 |
| Height (cm) | 167.6 ± 5.5 | 167.6 ± 5.7 | .793 |
| Weight (kg) | 81.5 ± 19.2 | 80.7 ± 19.5 | .743 |
| BMI (kg/m²) | 29.0 ± 6.8 | 28.8 ± 6.8 | .784 |
| Systolic blood pressure (mmHg) | 119 ± 12 | 117 ± 11 | .408 |
| Diastolic blood pressure (mmHg) | 74 ± 10 | 73 ± 12 | .507 |
| Heart rate (bpm) | 74 ± 10 | 76 ± 10 | .157 |
| OGTT (75g) |  |  |  |
| Fasting plasma glucose (mmol/L) | 4.6 ± 0.5 | 4.6 ± 0.5 | .644 |
| 2 h plasma glucose (mmol/L) | 5.3 ± 1.6 | 5.6 ± 1.5 | .132 |
| Cholesterol (mmol/L) | 4.6 ± 1.0 | 4.6 ± 0.8 | .628 |
| HDL cholesterol (mmol/L) | 1.6 ± 0.3 | 1.6 ± 0.4 | .467 |
| Triglycerides (mmol/L) | 1.1 ± 0.5 | 1.2 ± 0.6 | .438 |
| Smoking | 7 (6.0) | 14 (10.1) | .257 |
| Parity |  |  | .383 |
| Nullipara | 68 (58.1) | 75 (54.3) |  |
| PCOS phenotype^a^ |  |  | .633 |
| Type I | 72 (61.5) | 79 (57.2) |  |
| Type II | 11 (9.4) | 14 (10.1) |  |
| Type III | 3 (2.6) | 8 (5.8) |  |
| Type IV | 31 (26.5) | 37 (26.8) |  |
| Metformin use at conception | 37 (31.6) | 48 (34.8) | .716 |
| Mode of conception |  |  | .050 |
| Spontaneously (%) | 61 (52.1) | 88 (63.8) |  |
| ART (%) | 56 (47.9) | 50 (36.2) |  |
| Weight gain in pregnancy (kg) | 10.1 ± 5.0 | 11.0 ± 6.0 | .295 |
| Pregnancy complications |  |  |  |
| Preterm birth | 10 (8.5) | 5 (3.6) | .084 |
| GDM | 28 (23.9) | 35 (25.4) | .899 |
| Preeclampsia | 10 (8.5) | 5 (3.6) | .084 |
| Placenta weight (g) | 660 ± 168 | 679 ± 151 | .377 |
| Birth anthropometrics |  |  |  |
| Weight z-score^b^ | -0.14 (-0.33 to 0.06) | 0.14 (-0.04 to 0.32) | .027 |
| Length z-score^b^ | -0.62 (-0.84 to -0.40) | -0.32 (-0.49 to -0.14) | .034 |
| Head circumference z-score^b^ | 0.13 (0.04 to 0.32) | 0.18 (-0.01 to 0.37) | .710 |
| Gender; male/female | 54 (46.2) / 63 (53.8) | 75 (54.3) / 63 (45.7) | .357 |
| Breastfeeding |  |  |  |
| Exclusive (months) | 4.3 ± 2.9 | 4.6 ± 3.1 | .509 |
| Total (months) | 9.1 ± 4.1 | 8.9 ± 4.5 | .807 |

S3 Table: Maternal characteristics early in pregnancy at inclusion in the PregMet-study, pregnancy outcomes, birth anthropometrics and breastfeeding in participants and non-participants

Data presented as mean ± standard deviation, numbers (%) or mean (95% Confidence Interval) as appropriate

BMI: body mass index calculated from the formula weight (kg)/ height (m)2; bpm: beats per minute; OGTT: oral glucose tolerance test; HDL: High density lipoprotein; ART: assisted reproductive therapy comprises ovulation induction, In Vitro Fertilization and Intracytoplasmic Sperm Injection; GDM: gestational diabetes mellitus defined as fasting plasma glucose higher than 7·0 mmol/L and/or 2-h serum glucose higher than 7·8 mmol/L after an 75 g oral glucose tolerance test

^a^PCOS phenotypes: Type 1: Polycystic ovaries (PCO) + hyperandrogenism + oligoamenorrhea; Type II: PCO + hyperandrogenism; Type III: hyperandrogenism + oligoamenorrhea; Type IV: PCO + oligoamenorrhea

^b^z-scores were calculated according to gender and age from a Norwegian reference population (29,30)
