# Supplementary material for: Maternal PCOS status and metformin in pregnancy: Steroid hormones in 5–10 years old children from the PregMet randomized controlled study
Source: PLoS One. 2021 Sep 9;16(9):e0257186. doi: 10.1371/journal.pone.0257186 (PMC8428669; doi:10.1371/journal.pone.0257186)
Supplement: S4 Table — (DOCX) [file pone.0257186.s004.docx]

| Children of both sexes |  |  |
| --- | --- | --- |
|  | Placebo, n=54  Median (IQR) | Metformin, n=63  Median (IQR) |
| Androstenedione nmol/L | 0.51 (0.35) | 0.50 (0.35) |
| Testosterone nmol/L | 0.12 (0.08) | 0.12 (0.11) |
| SHBG (nmol/L) | 98 (50) | 106 (38) |
| Cortisol (nmol/L) | 190 (106) | 213 (110) |
| 17-OH-progesterone (nmol/L) | 0.60 (0.37) | 0.70 (0.53) |
| 11-deoxycortisol (nmol/L) | 0.87 (0.72) | 1.13 (1.18) |
| Free testosterone | 0.13 (0.12) | 0.10 (0.12) |
| Boys |  |  |
|  | Placebo, n=24 | Metformin, n=31 |
|  | Median (IQR) | Median (IQR) |
| Androstenedione nmol/L | 0.36 (0.43) | 0.47 (0.30) |
| Testosterone nmol/L | 0.10 (1.15) | 0.11 (0.09) |
| SHBG (nmol/L) | 131 (64) | 101 (39) |
| Cortisol (nmol/L) | 186 (102) | 213 (124) |
| 17-OH-progesterone (nmol/L) | 0.51 (0.37) | 0.61 (0.71) |
| 11-deoxycortisol (nmol/L) | 0.75 (0.35) | 1.24 (1.98) |
| Free testosterone | 0.08 (0.10) | 0.09 (0.10) |
| Girls |  |  |
|  | Placebo, n= 30 | Metformin, n=32 |
|  | Median (IQR) | Median (IQR) |
| Androstenedione nmol/L¤ | 0.51 (0.34) | 0.55 (0.46) |
| Testosterone nmol/L¤ | 0.15 (0.07) | 0.12 (0.12) |
| SHBG (nmol/L) | 90 (24) | 107 (43) |
| Cortisol (nmol/L)¤ | 194 (109) | 215 (112) |
| 17-OH-progesterone (nmol/L)¤ | 0.65 (0.39) | 0.70 (0.51) |
| 11-deoxycortisol (nmol/L)¤ | 0.98 (0.74) | 1.09 (0.75) |
| Free testosterone | 0.18 (0.12) | 0.13 (0.17) |

**S4 Table. Hormonal levels in placebo and metformin exposed children including children of both sexes, boys and girls**

CI: confidence interval; SHBG: sex hormone binding globulin; IQR: Interquartile Range
