# Supplementary material for: Maternal PCOS status and metformin in pregnancy: Steroid hormones in 5–10 years old children from the PregMet randomized controlled study
Source: PLoS One. 2021 Sep 9;16(9):e0257186. doi: 10.1371/journal.pone.0257186 (PMC8428669; doi:10.1371/journal.pone.0257186)
Supplement: S5 Table — (DOCX) [file pone.0257186.s005.docx]

| **Children of both sexes** |  |  |  |  |
| --- | --- | --- | --- | --- |
|  | The placebo effect  Unadjusted  Mean (95% CI) | p | The placebo effect  Adjusted for offspring BMI z-score  Mean (95% CI) | p |
| Androstenedione z-score | 0.53 (0.27 to 0.79) | .0001 | 0.51 (0.24 to 0.77) | .0004 |
| Testosterone z-score | 0.53 (0.29 to 0.77) | <.0001 | 0.45 (0.22 to 0.69) | <.0001 |
| SHBG z-score | -0.28 (-0.55 to 0.00) | .051 | -0.15 (-0.40 to 0.10) | .225 |
| Cortisol z-score | 0.34 (0.08 to 0.61) | .012 | 0.41 (0.15 to 0.67) | .003 |
| 17-OH-progesterone z-score | 0.42 (0.15 to 0.69) | .003 | 0.40 (0.12 to 0.67) | .006 |
| 11-deoxycortisol z-score | 0.13 (-0.11 to 0.37) | .269 | 0.14 (-0.11 to 0.39) | .265 |
| Free testosterone z-score | 0.67 (0.38 to 0.96) | <.0001 | 0.53 (0.28 to 0.78) | <.0001 |
| **Boys** |  |  |  |  |
|  | The placebo effect  Unadjusted  Mean (95% CI) | p | The placebo effect  Adjusted for offspring BMI z-score  Mean (95% CI) | p |
| Androstenedione z-score | 0.27 (-0.15 to 0.69) | .195 | 0.25 (-0.18 to 0.67) | .236 |
| Testosterone z-score | 0.25 (-0.20 to 0.70) | .264 | 0.19 (-0.21 to 0.60) | .332 |
| SHBG z-score | 0.03 (-0.45 to 0.51) | .905 | 0.10 (-0.32 to 0.51) | .641 |
| Cortisol z-score | 0.51 (0.17 to 0.84) | .005 | 0.53 (0.19 to 0.87) | .004 |
| 17-OH-progesterone z-score | 0.40 (-0.07 to 0.87) | .089 | 0.35 (-0.09 to 0.79) | .109 |
| 11-deoxycortisol z-score | 0.08 (-0.31 to 0.47) | .670 | 0.06 (-0.33 to 0.45) | .757 |
| Free testosterone z-score | 0.25 (-0.23 to 0.73) | .292 | 0.16 (-0.20 to 0.53) | .352 |
| **Girls** |  |  |  |  |
|  | The placebo effect  Mean (95% CI) | p | The placebo effect  Adjusted for offspring BMI z-score  Mean (95% CI) | P |
| Androstenedione z-score | 0.73 (0.41 to 1.06) | <.0001 | 0.74 (0.39 to 1.10) | .0002 |
| Testosterone z-score | 0.76 (0.51 to 1.00) | <.0001 | 0.71 (0.46 to 0.97) | <.0001 |
| SHBG z-score | -0.51(-0.83 to-0.19) | .003 | -0.38 (-0.68 to -0.07) | .091 |
| Cortisol z-score | 0.21 (-0.19 to 0.61) | .291 | 0.32 (-0.09 to 0.73) | .125 |
| 17-OH-progesterone z-score | 0.44 (0.11 to 0.76) | .010 | 0.48 (0.14 to 0.83) | .008 |
| 11-deoxycortisol z-score | 0.18 (-0.14 to 0.49) | .291 | 0.24 (-0.10 to 0.57) | .155 |
| Free testosterone z-score | 0.99 (0.67 to 1.32) | <.0001 | 0.87 (0.55 to 1.18) | <.0001 |
|  |  |  |  |  |

**S5 Table. Effect of maternal PCOS on steroid hormones in children, unadjusted, and adjusted for children BMI z-score**

CI: confidence interval; SHBG: sex hormone binding globulin;
