# Supplementary material for: Maternal PCOS status and metformin in pregnancy: Steroid hormones in 5–10 years old children from the PregMet randomized controlled study
Source: PLoS One. 2021 Sep 9;16(9):e0257186. doi: 10.1371/journal.pone.0257186 (PMC8428669; doi:10.1371/journal.pone.0257186)
Supplement: S6 Table — (DOCX) [file pone.0257186.s006.docx]

| **Children of both sexes** |  |  |  |  |
| --- | --- | --- | --- | --- |
|  | The metformin effect  Unadjusted  Δ M-P (95% CI) | p | The metformin effect  Adjusted for BMI z-score  Δ M-P (95% CI) | p |
| Androstenedione z-score | 0.13 (-0.23 to 0.49) | .470 | 0.08 (-0.28 to 0.43) | .675 |
| Testosterone z-score | 0.00 (-0.33 to 0.33) | .995 | -0.09 (-040 to 0.21) | .546 |
| SHBG z-score | 0.01 (-0.36 to 0.34) | .973 | 0.10 (-0.20 to 0.41) | .492 |
| Cortisol z-score | 0.35 (-0.03 to 0.72) | .070 | 0.39 (0.01 to 0.77) | .044 |
| 17-OH-progesterone z-score | 0.41 (0.08 to 0.74) | .015 | 0.40 (0.06 to 0.74) | .022 |
| 11-deoxycortisol z-score | 0.32 (-0.01 to 0.66) | .056 | 0.30 (-0.03 to 0.64) | .077 |
| Free testosterone z-score | -0.04(-0.44 to 0.36) | .841 | -0.20 (-0.52 to 0.13) | .230 |
| **Boys** |  |  |  |  |
|  | Unadjusted  Δ M-P (95% CI) | p | Adjusted for BMI z-score  Δ M-P (95% CI) | p |
| Androstenedione z-score | 0.46 (-0.02 to 0.95) | .058 | 0.35 (-0.13 to 0.82) | .151 |
| Testosterone z-score | 0.29 (-0.23 to 0.81) | .268 | 0.08 (-0.39 to 0.56) | .732 |
| SHBG z-score | -0.46 (-0.99 to 0.08) | .096 | -0.25 (-0.74 to 0.24) | .316 |
| Cortisol z-score | 0.31 (-0.27 to 0.89) | .284 | 0.41 (-0.18 to 0.99) | .171 |
| 17-OH-progesterone z-score | 0.53 (-0.00 to 1.06) | .052 | 0.49 (-0.06 to 1.04) | .080 |
| 11-deoxycortisol z-score | 0.65 (0.14 to 1.17) | .014 | 0.62 (0.08 to 1.15) | .025 |
| Free testosterone z-score | 0.42 (-0.13 to 0.97) | .133 | 0.13 (-0.30 to 0.56) | .551 |
| **Girls** |  |  |  |  |
|  | Unadjusted  Δ M-P (95% CI) | p | Adjusted for BMI z-score  Δ M-P (95% CI) | p |
| Androstenedione z-score | -0.15 (-0.68 to 0.38) | .580 | -0.15 (-0.68 to 0.38) | .575 |
| Testosterone z-score | -0.23 (-0.66 to 0.19) | .280 | -0.25 (-0.65 to 0.15) | .223 |
| SHBG z-score | 0.37 (-0.08 to 0.83) | .104 | 0.40 (0.15 to 0.79) | .042 |
| Cortisol z-score | 0.35 (-0.15 to 0.86) | .165 | 0.37 (-0.15 to 0.89) | .160 |
| 17-OH-progesterone z-score | 0.30 (-0.13 to 0.73) | .162 | 0.30 (-0.15 to 0.74) | .188 |
| 11-deoxycortisol z-score | 0.01 (-0.41 to 0.44) | .952 | 0.00 (-0.43 to 0.43) | 1.0 |
| Free testosterone z-score | -0.40 (-0.96 to 0.16) | .157 | -0.46 (-0.93 to 0.02) | .062 |

**S6 Table. Metformin effect on steroid hormones in children from the PregMet study, unadjusted, and adjusted for BMI z-score**

No p-values were statistically significant after Holm-Bonferroni adjustment

Δ M-P expresses the metformin effect on hormone mean z-scores in offspring born to mothers with PCOS

CI: confidence interval; SHBG: sex hormone binding globulin;
